# Visual risk factors for falls in older adults: a case-control study

**DOI:** 10.1186/s12877-022-02784-3

**Published:** 2022-02-17

**Authors:** Jignasa Mehta, Gabriela Czanner, Simon Harding, David Newsham, Jude Robinson

**Affiliations:** 1 School of Health Sciences, Institute of Population Health, University of Liverpool, Thompson Yates Building, L69 3GB Liverpool, UK; 2 School of Computer Science and Mathematics, Faculty of Engineering and Technology, Liverpool John Moores University, Liverpool, UK; 3 Faculty of Informatics and Information Technology, Slovak University of Technology, Bratislava, Slovakia; 4 Department of Eye and Vision Science, Institute of Life Course and Medical Sciences, University of Liverpool, Liverpool, UK; 5 St. Pauls Eye Unit, Liverpool University Hospitals NHS Foundation Trust, Liverpool, UK; 6 School of Social and Political Sciences, University of Glasgow, Glasgow, UK

**Keywords:** Falls, Vision, Depth perception, Contrast sensitivity, Social determinants

## Abstract

**Background:**

Falls are the second leading  cause of accidental deaths worldwide mainly in older people. Older people have poor vision and published evidence suggests that it is a risk factor for falls. Less than half of falls clinics assess vision as part of the multi-factorial assessment of older adults at risk of falls despite vision being an essential input for postural stability. The aim of our study was to investigate the relationship between all clinically assessed visual functions and falls amongst older adults in a prospective observational individually age-matched case control study.

**Methods:**

Visual acuity (VA), contrast sensitivity (CS), depth perception, binocular vision and binocular visual field were measured using routinely used clinical methods in falls participants (N = 83) and non-falls participants (N = 83). Data were also collected on socio-demographic factors, general health, number of medications, health quality, fear of falling and physical activity. Logistic regression analysis was carried out to determine key visual and non-visual risk factors for falls whilst adjusting for confounding covariates.

**Results:**

Older adults have an increased risk of experiencing a fall if they have reduced visual function (odds ratio (OR): 3.49, 1.64-7.45, p = 0.001), specifically impaired stereoacuity worse than 85” of arc (OR: 3.4, 1.20-9.69, p = 0.02) and reduced (by 0.15 log unit) high spatial frequency CS (18 cpd) (OR:1.40, 1.12-1.80, p = 0.003). Older adults with a hearing impairment are also at higher risk of falls (OR: 3.18, 95% CI: 1.36-7.40, p = 0.007). The risk decreases with living in a less deprived area (OR: 0.74, 0.64-0.86, <0.001), or socialising more out of the home (OR: 0.75, 0.60-0.93, p = 0.01).

**Conclusions:**

The combination of social, behavioural and biological determinants are significant predictors of a fall. The non-visual risk factors include older adults, living in deprived neighbourhoods, socialising less outside of the home and those who have a hearing impairment. Impaired functional visual measures; depth perception and contrast are significant visual risk factors for falls above visual acuity.

## Background

Falls are a significant public health issue and in 2017-2018 the Public Health Outcomes Framework in England reported around 220,160 emergency hospital admissions related to falls among patients aged 65 and over with 66% of these in older adults aged 80 and over [1]. The National Falls Prevention Coordination group recommend a whole system approach in their Falls and Fracture Consensus Statement [2] and the National Institute of Clinical Excellence (NICE) recommend that older adults have a multifactorial risk assessment including a vision assessment to reduce their risk of falls [2, 3]. A national survey of falls services reported that only 54% of professionals checked vision as part of their service [4].

Cohort studies have reported an increased risk of falls associated with a decline in visual acuity (VA) and contrast sensitivity (CS) [5–10]. However, there are few reports of a significant association between reduced depth perception and falls [6, 7]. Postural stability is achieved by adequate input from the visual, vestibular and somatosensory systems, processing of the information by the cortex, and finally an efficient motor response of the muscles, joints and reflexes [11]. A deficit in any of the sensory systems may affect balance and potentially put an individual at risk of falls [12]. The performance of the visual system is dependent on different visual functions operating at optimum level. It is not judged on simply resolving the smallest high contrast object at the furthest distance as in widely used standard measures of VA, but additional measures of visual function such as contrast sensitivity, visual field, and binocular vision are also involved. There is no single published case-control study that compares these routine clinical measures of visual function with the risk of falls.

We understand that falls have a multi-factorial aetiology with some review studies reporting on a large number of risk factors [13–15]. However, there are limited studies examining the association between socioeconomic status and falls [16, 17]. Also It has been suggested that people with large networks have better health [18], however this has not been examined in relation to falls. Hence, we conducted a study to compare visual function measured with commonly used clinical assessment methods in older adults who had experienced a fall against age-matched older adults who had not experienced a fall in the previous 5 years whilst also adjusting for plausible non-visual risk factors. We hypothesise that there is a significant association between impaired visual function and falls.

## Methods

An observational case-control, individually age-matched study design was employed to investigate the association between deficient visual function and falls in older adults aged 60 years and over. A sample size of 166 participants in two groups was calculated using STATA/IC V13.1 adjusting for seven confounding variables and based on a clinically important 3 line ETDRS (Early Treatment Diabetic Retinopathy Study) visual acuity difference [19]. Falls participants were recruited from a falls clinic at the Royal Liverpool and Broadgreen University Hospital (Liverpool, UK) where they were referred either by their general practitioner or the emergency department.

A participant was included as a ‘falls participant’ if they had experienced a fall in line with the ProFaNE definition, ‘an unexpected event in which the participant comes to rest on the ground, floor, or lower-level’ [20]. Participants were seen within two months of their last fall and screened for cognitive impairment eligibility using the 6-item cognitive impairment test (6CIT) [21] and were excluded if they scored >7 points. Non-falls participants were included if they had not experienced a fall in the previous 5 years and recruited through the hospital research networks. The rationale for choosing no falls in a 5 year period for the control group was to reduce the possibility of older adults feeling the physical and psychological effect of a fall in a more recent time period. The study was conducted over a period of 18 months. As we aimed to investigate the association between visual risk factors and falls, we collected non-visual data for each participant that could be accounted for as potential confounding variables in the regression analyses. A total of 195 participants were enrolled, of which 8 participants (3 falls and 5 non-falls participants) did not meet the inclusion criteria, 6 participants (3 falls and 3 non-falls participants) withdrew their consent and 15 ‘falls’ participants were lost to follow up. However, we analysed complete data sets for 166 participants (as per the sample size calculation).

 The study was conducted according to the guidelines set out in the 1964 Declaration of Helsinki, and ethical approval was granted by the Harrow Research Ethics Committee (16/LO/2249). Written informed consent was obtained from all participants.

### Non-visual data

In addition to falls history, general health and medication, we noted additional demographic information such as socioeconomic status (SES), living arrangements and social activity. The SES was determined from the participants’ postal address and using the English Index of Multiple Deprivation (IMD) 2015, specifically the ‘Income Deprivation Affecting Older People Index’ (IDAOPI) [22]. Specific decile rankings from 1 (most deprived) to 10 (least deprived) were derived to facilitate analysis of differences in deprivation between falls and non-fall participants. Living arrangements included data on type of accommodation and the membership of the house. Social activity was determined by ascertaining the number of days each participant socialised in and out of the home.

Each participant completed three questionnaires: (1) the Falls Efficacy Scale-International (FES-I) for fear of falling [23], (2) Rapid Assessment of Physical Activity (RAPA) [24] and (3) the EQ-5D (EuroQoL-five dimensional questionnaire), a self-reported measure of health status [25]. The Timed Up To Go (TUTG) test was carried out as per the method outlined elsewhere [26].

### Visual data

**Fig. 1:**
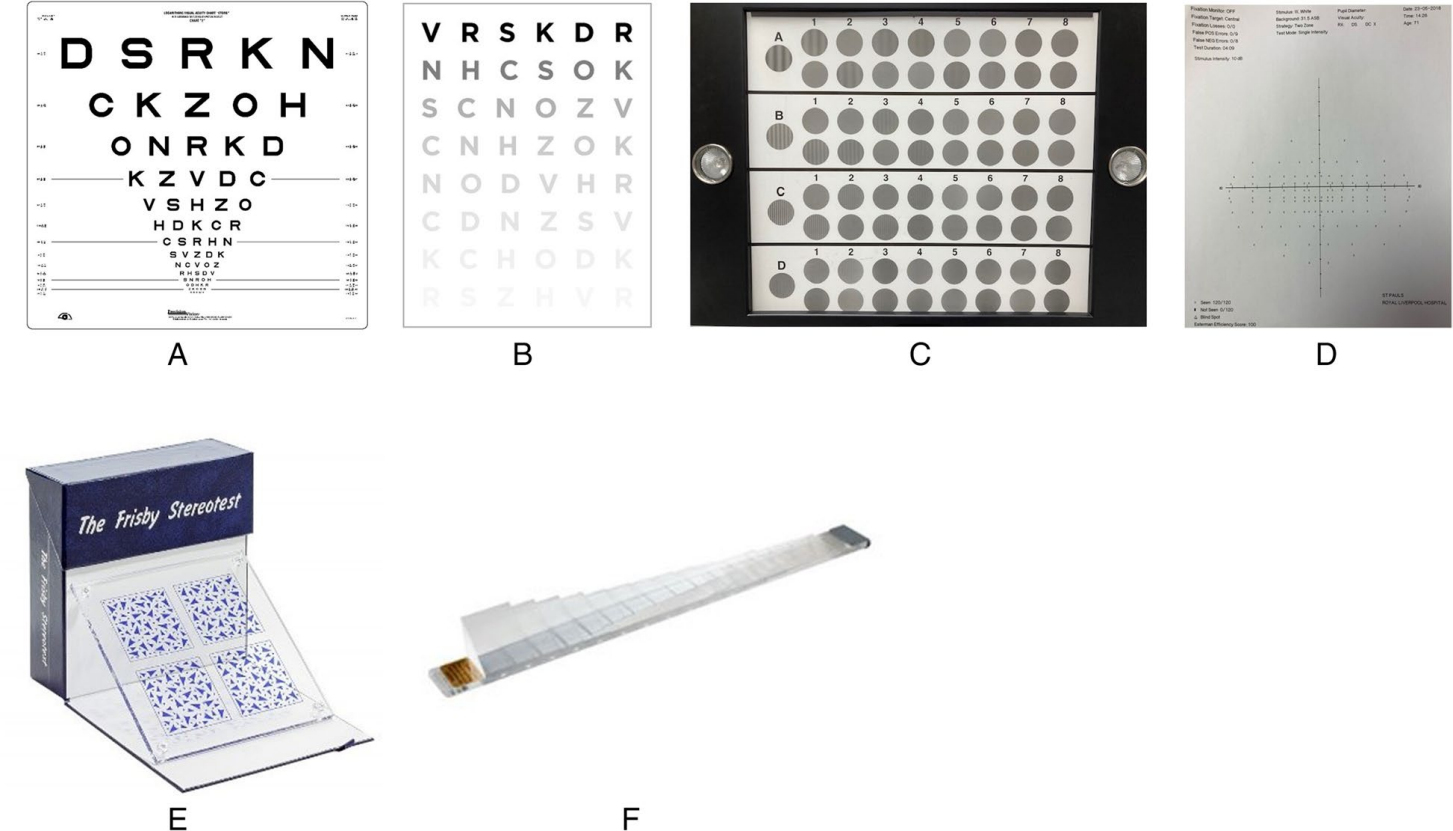
Clinical tests used to measure visual function (**A**-ETDRS Chart, **B**-Pelli-Robson, **C**-CSV-1000E, **D**-Binocular Esterman visual field, **E**-Frisby stereotest, **F**-Prism bar to measure prism fusion range

Each measure of visual function (Fig. 1) was measured by a single assessor (JM) in one room under the standard lighting conditions required for each of the tests. Participants were asked whether they wore single vision glasses, bifocals or varifocals. Habitual VA was measured in either eye using the ETDRS logMAR chart at 4 m [27]. Near VA was also tested with either eye at 40 cm using an ETDRS logMAR chart. CS was measured binocularly using two methods: Pelli-Robson (PR) and CSV 1000E. The PR chart is a routinely used test in clinics to measure CS. It is based on letters of fixed size reducing in contrast in steps of 0.15 log units from 0.00 to 2.25 log CS. The CSV-100E allows measurement of contrast at 3, 6, 12 and 18 cycles per degree (cpd). The Frisby test was used as per the instructions to measure stereoacuity in seconds of arc (depth perception). Prism fusion range measured with any habitual near and distance spectacle correction (in prism dioptres) assessed the ability of the participant to maintain fusion of an image through a range of convergence and divergence using base out and base in prisms respectively. Functional binocular visual field was measured with the Humphrey Field Analyzer binocular Esterman program.

### Statistical analysis

We employed SPSS 25 for the analyses. Normality was checked via Kolmogorov-Smirnov and Shapiro Wilk tests and accordingly applied parametric and non-parametric tests to compare matched and unmatched data between the two groups. For categorical data, the Chi-square test of association was used for comparing proportions of participants in each group. Stereo data were categorised as ‘no stereo’, ‘stereo outside normal limits, 110-600” of arc’ and ‘stereopsis within normal limits, 85-20” of arc’. A conservative estimate of 85” of arc was chosen as the ‘normal’ threshold based on normative data published in two small studies [28, 29].

All non-visual and visual function variables were initially analysed separately (univariate) using binary logistic regression. The outcome was ‘fall’ or ‘no fall’ and each of the significant explanatory variables were regarded as predictors and considered broadly under ‘non-visual’ and ‘visual’. If an explanatory variable was highly skewed, then this was log-transformed for the analyses.

**Fig. 2:**
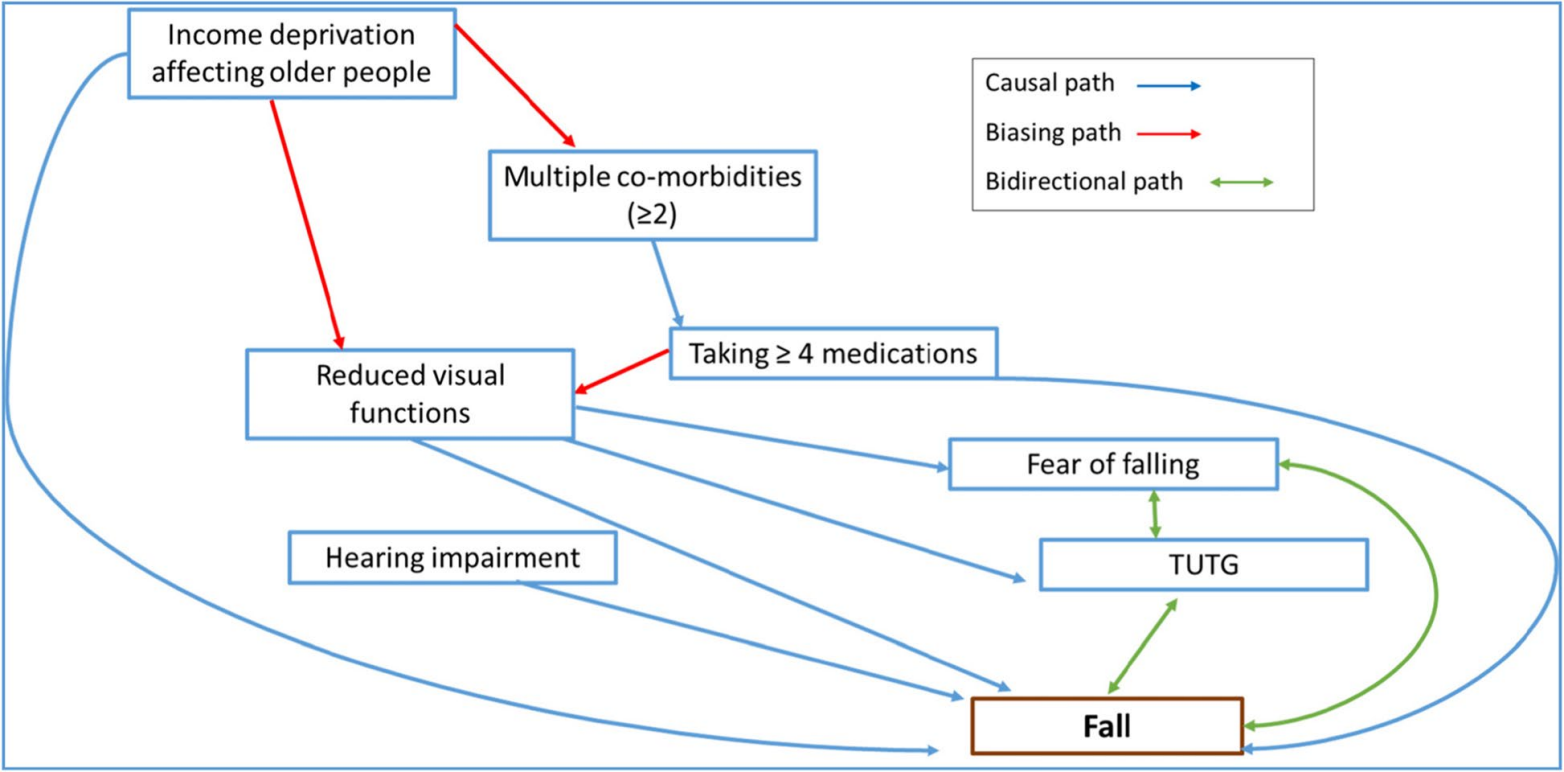
Directed Acyclic Graph demonstrating the causal pathways of a fall using the covariates measured in this study. All causal and biasing paths will be tested in the logistic regression model

Multivariable logistic regression models were constructed separately for non-visual (Model 1) and visual (Model 2) variables. A Directed Acyclic Graph (DAG) (Fig. 2) was employed to facilitate the appropriate selection of explanatory variables (visual and non-visual) for the multivariable logistic regression model to determine the association between reduced visual function and falls [30]. Fear of falling and TUTG were not entered into the model as deficits in both of these measures can be a consequence of the fall or a contributing risk factor and can be seen in the diagram as having a bi-directional arrow to illustrate the relationship, hence not satisfying the criteria of confounders [30]. An automated forward stepwise selection procedure was used to select the variables for the regression analysis. The ‘reduced visual functions’ variable was defined as an abnormal result in any of the visual function measures as follows:

- VA (6 m) ≥ +0.30 logMAR.
- PR with both eyes open (BEO) < 1.65 log units.
- CSV 1000E 3 cpm (BEO) ≤1.41 log units.
- CSV 1000E 6 cpd (BEO) ≤1.635 log units.
- CSV 1000E 12 cpd (BEO) ≤1.35 log units.
- CSV 1000E 18 cpd (BEO) ≤0.68 log units.
- Stereoacuity > 85” of arc log units..

Finally, a combination of non-visual and visual function variables was put together to identify the combination of specific non-visual and visual explanatory variables that predict falls risk (Model 3).

## Results

### Demographics

83 cases aged 60 years and over were recruited to each of the ‘falls’ and ‘non-falls’ (control) groups. The groups in this age-matched study had similar mean ages: falls group 72 years (SD 6.5), non-falls group 71 years (SD 6.5). Both groups had similar proportions of males and females (falls- male:female 35%:65%, non-falls- male:female 43%:57%, *p* = 0.3, Chi-square). However, within the falls group, there were significantly more female than male participants (*p* = 0.0008, Binomial exact test for proportions). Falls participants lived in areas that were 4 deciles (out of 10) more deprived compared to the postcode areas of the non-falls participants (*p* < 0.0001, Wilcoxon signed rank test). Table 1 illustrates the visual and non-visual data across the age matched groups of falls and non-falls participants.

**Table 1:** Non-visual and visual characteristics of the falls and non-falls participants

	Falls group (N=83)		Non-falls group (N=83)	
**Non-visual variables**				
Age mean (SD)	72 years ( 6.5)		71 years ( 6.5)	
Gender male:female % ratio	35%:65%		43%:57%	
IDAOPI decile Median [quartiles]	2* [1, 4]		6* [2, 8]	
Use of a walking aid %	Always: 16%* Occasionally: 26.5%*		Always: 4%* Occasionally: 12%*	
FES-I score median [quartiles]	31* [21,45]		18* [17,22]	
TUTG secs median [quartiles]	10* [7.25,13]		7* [6,8]	
Hearing impairment N(%)	32 (38.6%)*		11 (13.3%)*	
Number of pre-specified co-morbidities median [quartiles]	2 [1,2]*		1 [1,2]*	
Taking more than 4 medications N(%)	52 (62.7%)*		28 (33.7%)*	
Socialise out of the home no. of days median [quartiles]	5* [3,6]		6 [5,7]	
RAPA median [quartiles]	4 [3,6]*		6 [4,6]*	
EQ-5D VAS score mean (SD)	53 (21)*		84 (15.4)*	
**Visual variables**				
Better eye VA- 6m (mean±SD)	0.16 ±0.23*		0.07± 0.14*	
Better eye VA- 1/3m (mean±SD)	0.25±0.45*		0.14±0.16*	
Pelli Robson contrast sensitivity (log units) Both eyes- median [quartiles]	1.65* [1.35, 1.80]		1.65* [1.35, 1.80]	
CSV-1000E (log units) 3cpd Both eyes median [quartiles]	1.63* [1.49, 1.78]		1.63* [1.56, 1.78]	
CSV-1000E (log units) 6cpd Both eyes median [quartiles]	1.70* [1.38, 1.84]		1.84* [1.7, 1.99]	
CSV-1000E (log units) 12cpd Both eyes median [quartiles]	1.08* [0.61, 1.40]		1.40* [1.25, 1.54]	
CSV-1000E (log units) 18cpd Both eyes median [quartiles]	0.64* [0.17, 0.81]		0.96* [0.47, 1.10]	
Near prism fusion range median prism dioptres [quartiles]	**Base out** 20 [12, 30]	**Base in** 12 [8, 14]	**Base out** 30 [14, 35]	**Base in** 14 [10, 16]
Distance prism fusion range median prism dioptres [quartiles]	12^*^ [8, 20]	6 [4, 6]	20^*^ [12,25]	6 [4, 8]
Stereoacuity (Frisby) (seconds of arc)	85* [40, 170]		55* [30, 85]	
Binocular Esterman visual field “Fail”- 4 or more adjoining points missed N (%)	14 (17%)		10 (12%)	

* *p *<0.05, Univariate analysis

### Non-visual variables

Over a quarter of the participants (26.5%) had experienced five or more falls at the point of recruitment to the study. Further analysis by gender demonstrated that significantly more females (n = 42, 50.6%) than males (n = 17, 20.5%) had experienced ≥2 falls (p = 0.0001, Chi-square). The most common cause of the fall described by the participants was a trip (n = 36, 43%).

In the initial univariate logistic regression analysis, the significant non-visual risk factors for a fall included, IDAOPI, use of a walking aid ‘always’ or ‘occasionally’, having a high FES-I score, poor balance on TUTG, a hearing impairment, increased number of pre-specified co-morbidities, taking more than 4 medications, socialising out of the home, RAPA and the EQ-5D VAS score (all p < 0.05, univariate logistic regression analysis). There were no significant differences between the falls and non-falls participants in terms of the type of accommodation, whether they lived with others and the type of support that was available to them (all p > 0.05, Chi-square).

Multivariable logistic regression analysis of the significant non-visual risk factors demonstrated that individuals who lived in an area of high income deprivation, had a hearing impairment, participated less in social activity out of the home, and were less physically active were at a statistically significantly greater risk of having a fall (p < 0.05, logistic regression-Model 1, Table 2).

### Visual variables

Significantly more falls participants (41% vs. 17%) had separate pairs of single vision glasses for near and distance; more non-falls participants had just reading glasses (33% vs. 13%) (p = 0.001, Chi-Square). Five falls and three non-falls participants had VA in one eye that was either counting fingers, hand movements or perception/no perception of light. Falls participants had poorer distance VA (4.5 letters, 0.09 logMAR, p = 0.004 paired t-test) and near VA (5.5 letters, 0.11 logMAR, p = 0.04, paired t-test) than the non-falls participants when the better eye VA was compared across both groups. Significantly more falls participants (n = 15, 18%) had VA equal to or worse than +0.30 logMAR in their better eye compared to the non-falls participants (n = 5, 6%) (p = 0.018, Chi-square).

Of the total sample, stereoacuity was undetectable in 11 falls and 10 non-falls participants. These individuals were considered to be stereo-deficient and included in the analysis as a separate group. In the falls participants, the main cause of being stereo-deficient was poor monocular acuity either due to retinal disease (n = 8), cataract (n = 1), amblyopia (n = 1) or poor binocular control (n = 1). In the non-falls participants, the causes for stereo-deficiency were similar although more individuals were amblyopic (n = 5) (retinal disease, n = 4 and ocular motility issue, n = 1). There was no significant difference between the groups in the proportions of individuals failing the binocular Esterman visual field test (p > 0.05, Fisher’s exact test). The base out range for distance fixation was the only fusional amplitude significantly reduced in the falls participants (20^∆^ vs 12^∆^ median, p = 0.012, Wilcoxon signed-rank test). All other visual function covariates were significant risk factors following a univariate logistic regression analysis (p < 0.05). Multivariable logistic regression analysis of the significant visual covariates identified CS measured at 18 cpd and stereoacuity outside of the normal limits (worse than 85” of arc) as significant risk factors for falls (p < 0.05, logistic regression-Model 2, Table 2).

**Table 2:** Model 1- multivariable model of significant non-visual risk factors, Model 2- multivariable logistic regression of visual risk factors. Model 3-combined multivariable regression model of significant non-visual and visual function variables as covariates from Models 1& 2( p < 0.05)

**Variable**	***β***	**se(** ***β*** **)**	**Odds ratio**	**95% CI**	**p-value**
**Model 1- Significant non-visual risk factors**					
**Income affecting** **deprivation in older** **people (IDAOPI)**	-0.29	0.07	0.75	0.66-0.86	<0.001
**Hearing impairment**	1.26	0.43	3.51	1.50-8.22	0.004
**Socialising out of the** **home**	-0.23	0.12	0.78	0.63-0.99	0.04
**RAPA**	-0.41	0.14	0.66	0.51-0.87	0.003
**Model 2- Significant visual risk factors**					
**CSV-1000E** **Both eyes** **18 cpd**	-2.81	0.66	0.06	0.02-0.22	<0.001
**Stereoacuity** **outside of normal** **limits (110”-600”)**	0.90	0.45	2.47	1.02-5.98	0.045
**Model 3- Significant visual and non-visual risk factors**					
**Income deprivation** **affecting older** **people index**	-0.30	0.07	0.74	0.64-0.86	<0.001
**Hearing impairment**	1.24	0.46	3.44	1.39-8.54	0.008
**Socialising out of the** **home**	-0.29	0.11	0.75	0.60-0.93	0.01
**CSV-1000E log** **units- Both eyes** **18 cpd median**	-2.23	0.76	0.11	0.02-0.48	0.003
**Stereoacuity outside** **of normal limits** **(110”-600”)**	1.23	0.53	3.4	1.20-9.69	0.02

### Multivariable model for the association between falls and reduced visual function

Based on the DAG considerations (Fig. 2); ‘reduced visual function’, ‘IDAOPI’, ‘no. of pre-specified comorbidities’, ‘taking 4 or more medications’ and ‘hearing impairment’ were entered into a multivariable logistic regression model to test the association of falls with reduced measures of visual function. Income affecting deprivation in older people and both sensory impairments: hearing and vision, were significant risk factors of a fall (p < 0.05, logistic regression, Table 3).

**Table 3:** Multivariable logistic regression model of significant risk factors. Forward selection procedure selected from these covariates: reduced visual function, income affecting deprivation in older people, no. of prespecified comorbidities, taking 4 or more medications and hearing impairment

Variable	*β*	se(*β*)	Odds ratio	95% CI	p-value
**Income affecting** **deprivation in older** **people**	-0.25	0.06	0.78	0.69-0.88	<0.001
**Hearing impairment**	1.16	0.43	3.18	1.36-7.40	0.007
**Reduced visual** **function** **(VA or CS or stereo)**	1.25	0.39	3.49	1.64-7.45	0.001

Finally, the association of falls with significant visual risk factors, impaired hearing and income deprivation were tested in a third multivariable model. For this, a combined model of the significant covariates from Models 1 and 2 (Tables 1 and 2) was fitted to the data. Impairments in CS, depth perception and hearing along with income deprivation and infrequent social activity outside of the home remained as significant risk factors for falls in older adults (p < 0.05, logistic regression-Model 3, Table 2). The odds ratio (OR) provided for CS in Model 3 (Table 2) is based on a 1 log unit change, however we calculated the odds ratio for a 0.15 log unit reduction in CS which is typically the unit change for CS (OR= 1.40 (exp(-2.23*-0.15)) with 95% CI: (1.12-1.80) (1.12 = exp(log(0.48)*-0.15) and 1.80= exp(log(0.02)*-0.15)).

## Discussion

Our study found evidence that people who have had a recent fall, compared to age-matched people who have not, have significantly worse visual function, have a hearing impairment, and are more likely to live in a deprived area and to socialise less outside of the home.

We found that two measures of visual function, contrast sensitivity and stereoacuity were independently associated with having had a fall. If an older adult’s high spatial CS (18 cpd) deteriorates by 0.15 log units then we have shown that their odds of having a fall increases by 40% (OR=1.40, p = 0.003). In addition, our study demonstrates that there is a 3.4 times higher odds (OR=3.4, p = 0.04) of having a fall if an older adult has abnormal depth perception (worse than 85” of arc) compared to an age matched individual with normal depth perception.

Poor CS measured with the Melbourne Edge Test (MET) has been reported to be independently associated with postural instability [10], slower walking velocity, increased step width and reduced stride length [31]. There are fewer studies that have investigated CS at different spatial frequencies [8, 32]. Following adjustment for confounders, these studies reported CS measured at lower spatial frequencies of 3, 6 and 12 cpd [8] and 1.5 and 3 cpd [32] to be significant risk factors for falls. In contrast, we have demonstrated that CS measured at a higher spatial frequency (18 cpd) as a significant risk factor. Lord and Dayhew [7] reported higher stereoacuity levels ≥ 215” arc as a significant risk factor compared to our threshold of ≥110” arc.

CS at higher spatial frequencies relates to the individual’s ability to see fine detail at lower contrast levels. Impaired CS impacts on functional vision particularly when negotiating an environment full of varying contrasts and detail. In the case of falls, individuals are required to negotiate outdoor pavements, steps and varying levels of walking surfaces which may alter in contrast if they are not uniform and flat. Similarly, depth perception is utilised to judge distances and judging depth of kerbs and steps. Our study highlights the importance of testing CS and stereoacuity in older adults at risk of falls over visual acuity. In our study we have specifically identified a loss in CS at high spatial frequencies and the threshold for stereoacuity at which these become significant risk factors for falls.

Whilst it has been reported that individuals with reduced VA were 1.7 times more likely to experience a fall [33], it was statistically significant at the univariate level in our study but not in the multivariable analysis. A 4.5 letter difference in VA between the falls and non-falls participants although statistically significant, is not clinically important in terms of functional vision. However, the increased number of individuals with VA ≤ +0.30 logMAR in the better eye in the falls participants is of clinical importance. Hence, whilst CS and depth perception are more relevant measures of visual function in falls, it is important to determine that the VA is better than +0.30 logMAR in either eye in older adults at risk of falls.

The non-visual risk factors for a further fall in our study included a three times greater risk of having a fall in the presence of a hearing impairment. The association of hearing impairment and falls is consistent with other studies [34–36] and likely to be underestimated due to the self-reporting nature of the data. Furthermore, we have shown that living in a less deprived area and increasing social activity out of the home would reduce the risk of a further fall by 26% and 25% respectively. Older adults with dual sensory loss have been reported to have reduced social activity by the SHARE study (Survey of Health, Ageing and Retirement in Europe) and that the higher likelihood for social inactivity was attenuated by general health and socio-economic indicators [37]. These findings could be further explored with a qualitative study to understand the relationship between impaired sensory functions, socio-economic factors and social participation.

Limited studies have examined the impact of socioeconomic inequality specifically on falls in older adults [16, 17], and none in England. Marmot [38] highlighted the impact of an individual’s social and economic status on health inequalities which can arise due to many interactional factors such as housing, income, education, social isolation and disability. Multiple co-morbidities and polypharmacy are variables that contribute to the health profile of individuals from more disadvantaged areas. Whilst these were significant in the univariate analysis, it was older adults living in deprived areas that was a key risk factor for a fall in the adjusted analysis. Reducing health inequalities is a public health issue and Public Health England have published a resource for local government to implement specific interventions for different levels of risk, impact over time and across the life course to tackle health inequalities [39].

People with large social networks have better health [18] and it has been reported that loneliness is an independent risk factor for physical inactivity [40]. We found physical activity to be significant in the analysis where we included other non-visual risk factors; however, this was not significant in the final analysis which included visual and non-visual factors. A potential rationale for this is that physical activity can be related to socialising out of the home which was a significant risk factor in the final model.

Strengths of this study are the age matched study design, visual assessment shortly after the fall, and the adjustment in the models for confounders for demographic, general health, social and living arrangements and physical activity data. The main limitation is the cross-sectional design meaning that caution should be used in implying any causative effect of the variables on falls. Also, the self-reporting nature of the data (for example, hearing and reporting of falls) could lead to recall bias. Whilst in this study data on general health conditions was recorded and factored into the analysis, we suggest future work should aim to evaluate changes to the vestibular and somatosensory systems which are known to have an effect on postural stability [12].

Falls have a multi-factorial aetiology and in our study, we have specifically identified a combination of socio-economic (income deprivation affecting older people), behavioural (socialising out of the home) and biological (reduced contrast sensitivity, impaired depth perception and hearing) risk factors. Depending on the cause, these are potentially modifiable. For example, bilateral cataract surgery will improve contrast sensitivity and stereoacuity. Efforts on building healthy and inclusive communities to reduce deprivation and subsequently close the health gap and reduce inequalities lie with local government and authorities [39]. However, health care professionals can help modify the social and behavioural risk factors by engaging in social prescribing [41] whereby older adults are signposted to non-clinical services and by promoting public health messaging.

Our study highlights the importance of a whole system approach, addressing socioeconomic, biological and behavioural issues to reduce the number of falls in older adults. We need to look beyond visual acuity in the multifactorial assessment of older adults at risk of falls and include an assessment of CS and stereoacuity as functional measures of vision. Older people with visual deficits should be referred for investigation and interventions to manage correctable visual impairments. Furthermore, healthcare professionals are ideally positioned to engage in behaviour change conversations to improve the social and behavioural determinants of falls in older adults.

## Data Availability

The datasets used and/or analysed during the current study are available from the corresponding author on reasonable request.
